# Development and Progression of Bovine Respiratory Disease Measured Using Clinical Respiratory Scoring and Thoracic Ultrasonography in Preweaned Calves on Dairy Farms in the United Kingdom: A Prospective Cohort Study

**DOI:** 10.3390/ani15030360

**Published:** 2025-01-27

**Authors:** George Lindley, Nicola Blackie, D. Claire Wathes, Richard E. Booth

**Affiliations:** Pathobiology and Population Sciences, Royal Veterinary College, Hawkshead Lane, Hatfield, Hertfordshire AL9 7TA, UK; nblackie@rvc.ac.uk (N.B.); rbooth@rvc.ac.uk (R.E.B.)

**Keywords:** respiratory disease, pneumonia, calf, ultrasound

## Abstract

The respiratory health of preweaned calves is an important determinant of their health, welfare, and future performance. This prospective cohort study measured bovine respiratory disease (BRD) on 16 dairy farms, including 476 calves in South-west England. Wisconsin and California respiratory scoring and thoracic ultrasonography were performed repeatedly at 7 ± 0.89 day intervals (mean ± SD) at 0–56 days of age (n = 3344 examinations). Cases were localized to the upper or lower respiratory tract, or both, and classified as new, repeat, or chronic. Prevalence and incidence were calculated. Multivariate modeling of factors associated with repeated measurements was performed. Increasing age (OR = 1.05, 95% CI 1.04–1.06) and fecal score (Score 2, OR = 1.78, 95% CI 1.14–2.77) were associated with a lower odds of a healthy BRD subtype, whereas increasing serum total protein (OR = 0.97, 95% CI 0.96–0.99) was protective. Older (OR 1.08, 95% CI 1.06–1.09), male (OR 1.69, 95% CI 1.01–2.84) calves with elevated Wisconsin respiratory scores (≥5, OR 5.61, 95% CI 3.38–9.30) were more likely to have elevated thoracic ultrasound scores. BRD remains common in calves born in UK dairy herds, requiring precise identification and management if preweaning health is to be optimized.

## Simple Summary

Bovine respiratory disease (BRD) in calves is common, but variable clinical signs throughout the disease process make appropriate treatment and prevention strategies difficult. This study sought to combine diagnostic techniques to describe the course of disease within UK dairy herds and factors associated with its progression. Preweaned calves were repeatedly measured between birth and weaning using both clinical scoring methods and lung ultrasonography to characterize the onset and progression of respiratory disease. Ultrasonographic lesions without associated clinical signs were more common than clinical pneumonia, but some calves with clinical signs were sonogaphically normal. The prevalence of new, repeat, and chronic cases of BRD varied dependent upon the diagnostic criteria and timing. Farm, age, immunity, gender, weight, and fecal score were significant predictors of disease subtype or lung ultrasound health. Accurate diagnosis and characterization of BRD allows precise management of respiratory well-being, which may be optimized by longitudinal assessments during the preweaning period.

## 1. Introduction

Bovine respiratory disease (BRD) represents one of the most common causes of morbidity and mortality in dairy calves globally [1,2,3]. It remains a key animal welfare issue and is the leading cause of antimicrobial usage in the preweaned calf population, although variable diagnostic criteria make comparisons between reported disease frequencies difficult [4]. For example, BRD prevalence measured using clinical signs or treatment records in preweaned calves in dairy herds has been recorded at between 8% and 23%, respectively [5,6], whereas when measured using a scoring system, a higher incidence of 45.9% was reported in the UK, with a range of 20.4 to 77.8% between 11 herds [7,8]. Measurements of the prevalence of lung consolidation have been similarly variable. Hinnant et al. [9] found evidence of consolidation in 63% of preweaned dairy calves assessed in two U.S. dairies and reported only a limited alignment of these measurements with clinical signs. Consolidation ≥ 1 cm depth in Belgian beef and dairy herds was 41.1% [4], whereas within spring-calving herds in Ireland, the median prevalence of lobular and lobar consolidation was in the range of 15–25% [2]. Baxter-Smith et al. [3] similarly measured a 15.3% prevalence of lobular/lobar consolidation on seven Scottish dairies, however examinations were limited to the caudal lung lobes and are therefore a likely underestimation.

Due to its multifactorial nature, varied disease presentations, and the lack of a gold-standard diagnostic, the measurement of BRD antemortem is challenging [10,11]. Clinical signs include nasal discharge, cough, pyrexia, respiratory changes, or ocular signs [12]. When these symptoms are combined, their standardized assessment is possible using clinical respiratory scoring (RS), such as the California or Wisconsin methods [13,14]. Another diagnostic method for BRD is thoracic ultrasonography (TUS), which may be used to identify lung consolidation associated with bronchopneumonia. The diagnostic accuracy of RS compared to TUS for the identification of BRD is imperfect [11,15,16]. This may be because BRD is a dynamic disease process; hence, the development and persistence of clinical signs and lung consolidation may be discordant [9,17]. Between- and within-calf variations and poor inter-rater agreement for the recognition of clinical signs may complicate the identification of cases further [15,18]. Nonetheless, TUS and RS diagnostic methods can identify different pathologies associated with BRD, and their combined use allows the localization of respiratory disease to the upper or lower respiratory tract, or both. This presents an opportunity to allocate treatment with greater precision.

Self-limiting disease localized to the upper respiratory tract may not progress to bronchopneumonia and spontaneous recovery associated with a successful immune response may occur, potentially avoiding the requirement for antimicrobial therapy [18,19,20]. In contrast, subclinical pneumonia may be identified as cases of lung consolidation without simultaneous clinical symptoms identifiable using RS [21]. The management of this subgroup of animals is essential, since the presence of subclinical lung consolidation retains similar long-term consequences as cases of clinical disease, including reduced longevity, milk yield, reproductive performance, growth, and carcass quality [22,23,24,25,26,27,28]. Identification and treatment of these cases may lead to amelioration, although in some circumstances the benefits are only short-lived [17,29,30]. Crucially, the use of RS components is insensitive for the identification of lung consolidation [31,32]. This may be since the development of RS symptoms is variable during cases of BRD, or because studies of a cross-sectional design provide infrequent measurements, missing the interaction between CRS and TUS positivity [33]. Few available studies have investigated the former possibility, and of these, inferences have been based upon a low number of herds or calves, reducing the ability to generalize from the results [17,21,30]. The combined use of TUS and CRS has not been described within a UK setting, thus the progression and characterization of disease are unknown locally.

This study sought to address the important fact that the definition of BRD is not standardized and the lack of accepted “gold-standard” diagnostic tests and sampling frequencies has led to inconsistencies in the literature. We therefore measured the occurrence of different BRD subtypes in preweaned calves born in UK dairy herds weekly using both CRS and TUS methodologies in order to reaffirm if the factors associated with lung consolidation are consistent with those identified by studies with a cross-sectional design. The objectives of this study were: (i) To describe and characterize BRD disease occurrence using repeated measurements of TUS and CRS within populations of preweaned calves born in UK dairy herds and (ii) to investigate covariates associated with repeated measures of BRD subtype and lung consolidation measured during the preweaning period within populations of calves born in UK dairy herds.

## 2. Materials and Methods

All procedures performed in this study were prospectively approved by the Clinical Research Ethical Review Board at the Royal Veterinary College (URN: 20222125-2).

### 2.1. Farm Selection and Calf Enrolment

A convenience sample of 16 commercial dairy farms located throughout South-west England was selected based on their location, calving pattern, and willingness to participate. During weekly visits between October 2022 and October 2023, dairy and dairy x beef calves born on each of the study farms and aged between 1 and 7 days were eligible to be enrolled.

A target sample size of 24–30 calves per farm was chosen based on an estimation using Epitools [34]. An approximate prevalence of BRD was estimated at 0.2, based on previous studies [2,3,5]. A precision of 0.1 was set to ensure an appropriate confidence interval width [35,36]. Confidence was set at 0.95. Additional considerations were recent similar study designs [2], likely losses to follow up, and the feasibility for data collection by a single researcher (GL) in order to eliminate inter-observer variation. At enrolment, blood was collected into a plain vacutainer for serum total protein (STP) analysis. Calves aged <1 day or >7 days at the first visit were excluded from this study. Initially, all eligible calves were enrolled, without a preference for calves destined for dairy or beef production. On certain farms, if calves destined for beef production departed the study prior to the end of the follow-up period, replacement heifers were enrolled to ensure an adequate number of calves remained under study during the later stages of the preweaning period.

### 2.2. Collection of Health and Morphometric Data

Calves received repeat examinations on an approximately weekly basis until they reached 56 days of age, departure from the herd, weaning, or death, whichever occurred earliest. All examinations were performed by the same researcher. At enrolment, calf gender and breed were recorded, and blood was collected via jugular venipuncture into a plain vacutainer and kept chilled until arrival at the laboratory, when it was centrifuged at 7400× *g* for 60 s. The serum was separated and, following calibration using distilled water, an aliquot was examined using a light refractometer (Burtons, Kent, UK) to determine the serum total protein concentration.

During each examination, the calves were restrained by halter and individual components of the Wisconsin respiratory score (WRS) [14] and the California respiratory score (CRS) [13] were recorded as described previously. This involved assessments of ocular discharge, nasal discharge, head tilt, ear droop, cough, breathing character, and rectal temperature, with the assignment of weighting for each parameter (Appendix A, Table A1). Thoracic ultrasonography was then performed using a portable ultrasound scanner with a linear probe (Easi-Scan Go, IMV Imaging, Bellshill, UK) set to a depth of 8 cm and a frequency in the range of 4.5–8.5 MHz, using 70% isopropyl alcohol as a transducing agent. Scanning was performed according to the University of Ghent quickscan TUS method (qTUS) by a single individual certified in the technique (GL), as described by Jourquin, et al. [37]. Briefly, the probe was passed in a single movement from the caudodorsal thorax in a cranioventral direction toward the elbow, until the heart base was visualized. The probe was then passed cranially, dorsal, then cranial to the heart until the internal thoracic artery and vein were visualized. The procedure was then performed on the other side of the thorax. In any circumstance where a lesion was identified, the advancement of the probe was stopped and the extent of the lesion measured before the examination was continued. A 6-point TUS score was used to interpret ultrasonographic findings as described by Ollivett et al. [38] (Appendix A, Table A2). Maximum depth of consolidation was measured in centimeters as a straight line perpendicular to the pleural line, using a 0.5 cm grid scale for measurement. Other lesions, such as pleural effusion, were not recorded. In addition to measures of respiratory disease, fecal (0–3) and hygiene scoring (3–9) were performed as described by McGuirk and Peek [14] and Kellermann et al. [39]. Heart girth was measured using a tape measure placed caudal to the forelimb in centimeters at approximately week 1, 1–7 days; week 5, 29–35 days; and week 8, 50–56 days, as described by Johnson et al. [40].

All the collected data were reported to farmers and their routine veterinarians within 24 h of each visit in order to inform treatment decisions. Any treatments were based on the advice of the private veterinarians and not the researchers. Records of BRD treatments and vaccinations were collected from each farm at the end of the study period and the former subdivided into non-steroidal anti-inflammatory drugs or antibiotics. No information regarding BRD pathogens was collected from any of the enrolled farms.

### 2.3. Disease Definitions

For both the California and the Wisconsin respiratory scores, a BRD positive score was defined as ≥5. In order to account for different severities of consolidation, two thresholds for the results of TUS scoring were analyzed: a score ≥ 2, equivalent to a minimum of lobular pneumonia with consolidation ≥ 1 cm^2^ (TUS2), and a score ≥ 3, equivalent to a minimum of lobar pneumonia with the consolidation of at least a single entire lung lobe (TUS3) (Appendix A, Table A2) [41].

The respiratory disease subtype was also determined by its classification into four categories of healthy, localized to the upper respiratory tract (URT); subclinical pneumonia, present in the lower respiratory tract (LRT); or clinical pneumonia, based on previous definitions (Table 1) [30,42]. Classification was performed separately using WRS and TUS, followed by CRS and TUS.

### 2.4. Measures of Disease Frequency

Since weaning occurred at 56 days (8 weeks) at most farms, the analysis of disease data was conducted until this point. Prevalence was calculated as all defined cases (new and existing) within the study population by week of age. Period prevalence was also calculated for two periods representing the first and second half of the follow-up duration: 1–28 days and 29–56 days. Incidence rates were calculated using calf-days at risk. Measures of disease incidence were calculated as described by Dohoo et al. [43]. Incidence rate was determined for the entire study period and by examination number. The number of calf-days at risk was calculated exactly using the age of each calf at each examination. For examinations during weeks 2–8, the total calf days during the previous examination week was subtracted to determine the calf-days at risk for that week only. Only new cases during each week of analysis were eligible for inclusion in the calculations of incidence. For incidence calculation with each diagnostic method, calves with a positive diagnosis were censored such that they were ineligible for later incidence estimations, as were individuals with missing data during the week of analysis. Incidence rate was defined as the total number of new cases divided by the number of calf-days at risk. This rate was then multiplied by 100 to report cases per 100 calf-days at risk. Incidence risk was calculated for each week as the total number of new cases during the week of analysis, divided by the number of calves at risk.

**Figure 1:**
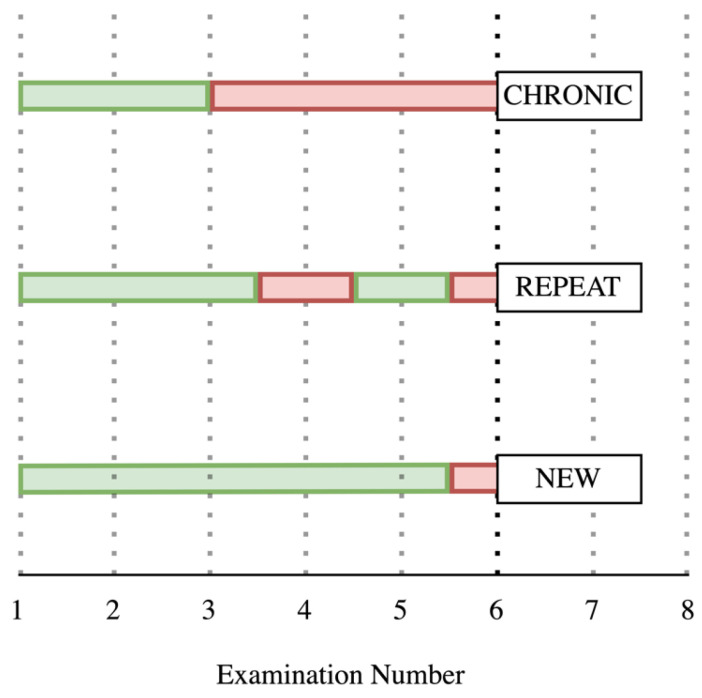
Criteria used to define new, repeat, and chronic cases of bovine respiratory disease, using either Wisconsin or California respiratory scoring (≥5), or a thoracic ultrasound score threshold of ≥2 (TUS2) or ≥3 (TUS3). Repeated examinations at weekly intervals are represented by the vertical dotted lines, whilst green indicates a below-threshold score in an individual calf and red an above-threshold score.

The percentages of new, repeat, and chronic cases were determined for each diagnostic method. Firstly, case definitions were produced (Figure 1). For each disease measure (WRS, CRS, TUS2, and TUS3), a new case was defined as the occurrence of an above-threshold score measured in an individual with no previous above-threshold score for that specific disease measure. A repeat case was defined as an above-threshold score followed by at least one clinically normal score and then a subsequent above-threshold score. Finally, a chronic case was defined as an above-threshold score measured in an individual for a duration of ≥21 days (equivalent to a minimum of three consecutive examinations). Analysis was performed during the entire study period, as well as for 21-day periods. Any calves that missed examinations during each analysis were excluded.

### 2.5. Data Analysis

Data were stored, cleaned, and analyzed using Excel (Microsoft Corp., Redmond, WA, USA) and R [44]. The age at which each examination was performed was used to categorize the examinations by week (i.e., week 1, 1–7 days; week 2, 8–14 days; week 3, 15–21 days; week 4, 22–28 days; week 5, 29–35 days; week 6, 36–42 days; week 7, 43–49 days; and week 8, 50–56 days).

Descriptive statistics of the study population were summarized according to calf sex, breed, losses to follow up, and treatments provided. Disease measurements at each examination were dichotomized using the aforementioned cut-offs to describe the percentage of healthy and diseased animals during each time period using each measure. Predicted calf weight was estimated using all available heart girth measurements using the formula described by Heinrichs et al. [45]. Confidence intervals for incidence measures were calculated using MedCalc (MedCalc Software version 23.1.3, Ostend, Belgium) by the substitution method as described by Daly [46], whereas for prevalence measures, it was determined using the online tool “Epitools” by Wald approximation [34].

Binary logistic regression modeling was performed to investigate covariates associated with the outcome measures of repeat or chronic cases identified using either TUS2 or TUS3 thresholds. Analysis was performed for chronic cases and then separately for repeat cases. Backward stepwise model building was performed with the covariates STP (g/L), farm, gender, purpose (dairy or beef production), treatment, average daily liveweight gain (ADG) (kg/d), and weight (kg).

To investigate factors associated with repeated measures of BRD subtype and TUS score during the first 56 days of life, generalized estimating equations (GEEs) were produced using the package “multgee” in R [47]. For the analysis of TUS score (0–5), an ordinal GEE was fitted with a logit link function and a uniform working correlation matrix. To ensure sufficient calves per analysis group, TUS scores were consolidated into three categories (category 1, TUS 0 and 1; category 2, TUS 2; and category 3, TUS ≥3). BRD subtype was analyzed based upon combined WRS and TUS2 definitions (see Table 1), and to facilitate the analysis, it was necessary to consolidate the outcome variable into 3 categories (1, healthy; 2, URT 3, subclinical, or clinical pneumonia). In addition, a nominal GEE was then constructed with a time exchangeable working correlation matrix. Marginalized local odds ratio estimates were performed simultaneously, with comparisons made between healthy and (sub)clinical pneumonia (i.e., both clinical and subclinical cases) and URT and (sub)clinical pneumonia. To avoid multicollinearity, both models were constructed in a forward stepwise approach with alpha set at 0.05. Each covariate was offered to the model, and during each step, the most significant covariate was retained based on the *p* value, until no additional significant predictors could be added. Model fit was verified by a comparison of model iterations using the Wald test, with models being retained if the improvement was significant (*p* < 0.01).

To investigate associations with the repeated outcome measure of maximum consolidation depth in centimeters over time, a linear mixed-effects model with an exchangeable correlation matrix was produced using the package “lme4” [48]. For this analysis, repeated measurements of consolidation depth in centimeters (level 1) were nested within calf (level 2) and within farm (level 3). This model was built using a backward elimination approach, whereby all eligible covariates for inclusion in the model were initially introduced. Thereafter, covariates were removed in a stepwise fashion based upon the *p* value, until all covariates remaining in the model were *p* < 0.20. Alpha was set at the level of 0.05.

For all repeated measures models, examination age and farm were initially forced into each model, with the covariates gender, breed, STP concentration (g/L as a continuous variable), fecal score, hygiene score, treatment, weight (kg), average daily liveweight gain (kg/d), and vaccination presented. The covariate WRS was transformed into a binary variable (WRS < 5 = 0, WRS ≥ 5 = 1) and included in analyses of the outcomes of consolidation depth and TUS score, but not BRD subtype due to confounding effects. Treatments (antimicrobial (AM) or non-steroidal anti-inflammatory (NSAID)) were transformed into an ordinal variable with a score in the range of 0–2 assigned (0 = no treatment, 1 = AM or NSAID, or 2 = AM + NSAID). Average daily liveweight gain (ADG) was calculated for all calves from the estimated bodyweight measurements collected during the first and final weeks of enrolment for all calves.

## 3. Results

### 3.1. Study Population

The characteristics of the 16 herds involved are described in Table 2. Data from a total of 476 calves from these herds were eligible for inclusion in the study, with a total of 3344 examinations performed repeatedly at weekly intervals of 7 days (SD = 0.89 days) (Table 3). For each time period, a small number of calves received a WRS and CRS, but not a TUS (range: 0–7 calves per time period; total = 24 exams). At enrolment, 23.3% (n = 111) of calves were male and 76.7% (n = 365) were female. Dairy breeds represented 56.9% (n = 271) of the population, of which 52.3% (n = 249) were Holstein or Holstein Friesian, 2.5% (n = 12) were Brown Swiss, and 2.1% (n = 10) were Norwegian Red. The dairy-cross beef population consisted of calves with Aberdeen Angus (26.9%, n = 128), Hereford (8.8%, n = 42), British Blue (6.1%, n = 29), or Limousin (1.3%, n = 6) sires. During the study period, 98 calves were lost to follow up due to departure from the farm of origin. Overall, 16 calves, representing 3.4% of the enrolled population, died during the study period. Although the data regarding the exact cause of death were unavailable, positive TUS2 and TUS3 scores were recorded in 14 (88%) and 12 (75%) of these cases, respectively, prior to death. A subset of 13 calves were weaned prior to 56 days of age and were therefore excluded from further analysis (Table 3). Mean serum total protein (STP) concentration was 62.6 g/L (SD = 10.13 g/L). A total of 61 calves (13%) had a poor STP < 51.0 g/L, whilst 48% (n = 227) had an excellent STP value ≥ 62.0 g/L when classified according to Lombard et al. [49]. Mean predicted bodyweight was 38.3 kg (SD = 7.15 kg), 49.7 kg (SD = 10.3 kg), and 61.3 kg (SD = 12.03 kg) during weeks 1, 5, and 8, respectively. The distribution of ADG was normal, with a mean value of 0.48 kg/d (SD = 0.17 kg/d).

A total of 55 calves received any AM and/or NSAID treatment during the study. On a weekly basis, the median number of calves receiving NSAID, AM therapy, or both was 7 (range: 6–14). When the number of doses was considered as a percentage of the calf population, between 0 and 3.5% of calves were administered NSAID or AM doses during the study period (Table 3). Calves were predominantly treated with either 15 mg/kg of amoxicillin (Betamox LA, Norbrook, Co Down, UK) or 20 mg/kg of florfenicol (Florkem, Ceva Animal Health Ltd., Buckinghamshire, UK), each accounting for 44% (n = 18) of total antibiotic usage. Associated treatment durations were short. Of calves receiving a first treatment, 45 involved a single dose, whereas 10 animals received 2–3 doses. Treatments were administered based on the estimated bodyweight in all circumstances and appeared to be consistent with product licensing. A total of 61 calves (13%) were vaccinated for BRD on three farms, with products varying by farm (farm 2, Bovilis^®^ Bovipast^®^ RSP (MSD Animal Health, Milton Keynes, UK); farm 5, Bovalto Respi Intranasal (Boehringer Ingelheim, Bracknell, UK); and farm 9, Bovilis^®^ INtranasal RSP^™^ Live (MSD Animal Health, Milton Keynes, UK)). This vaccinated subset included 75% (n = 49) females and 25% (n = 12) males. Holstein or Holstein Friesian breeds represented the majority (62%, n = 38), and intranasal formulations predominated (52%, n = 32).

### 3.2. Disease Frequency

#### 3.2.1. Prevalence and Incidence

**Figure 2:**
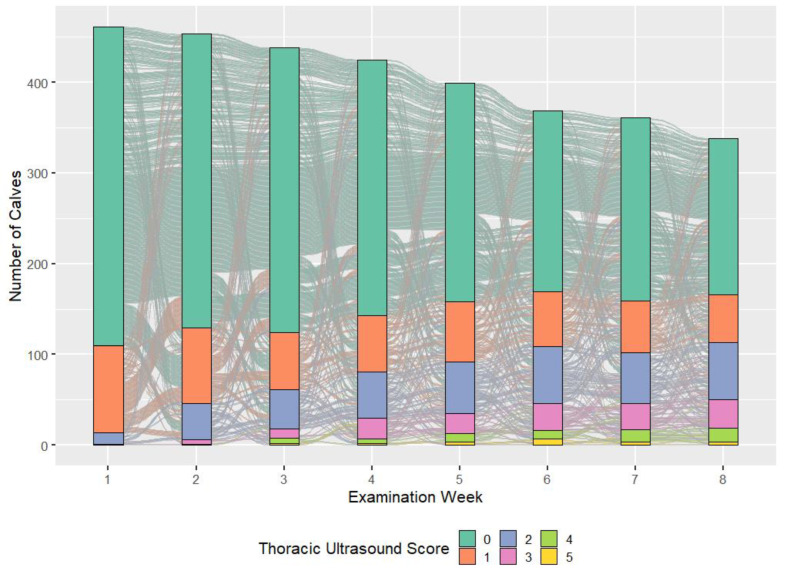
Alluvial plot of thoracic ultrasound score (TUS; 0–5), performed during repeated examinations of 470–349 calves during an 8-week period from birth. The vertical columns represent each examination week. The colors within each column correlate to a TUS score depicted in the key at the bottom of the figure. The individual lines passing between examination weeks represent individual calves, such that the TUS score can be followed during the study period.

The mean numbers of disease occurrences per animal for WRS, CRS, TUS2, and TUS3 were 0.38; 0.41; 1.61; and 0.64, respectively. As a percentage of all 3344 examinations performed, 4.6% (n = 156) and 5.1% (n = 172) included a positive WRS or CRS, whereas 18.3% (n = 639) and 7.2% (n = 239) included a positive TUS2 or TUS3, respectively. When assessed during days 1–28 and 29–56, the percentage of calves with a positive TUS2 score was 11.5% (n = 209) and 28.5% (n = 430), respectively. An increase in the percentage of calves with a positive TUS2 score was measured with increasing age (Figure 2, Table 4). The percentage of calves with a positive TUS3 score was comparatively low, only 3% (n = 55) and 12.1% (n = 184) during the same 1–28- and 29–56-day periods, respectively. In contrast, similar assessments using WRS detected positive scores in 3.6% (n = 66) and 6.2% (n = 93) of the examinations, whereas when measured using the CRS, similar proportions of 3.6% (n = 65) and 7.1% (n = 107) were above the cut-off. The incidence rate of new cases of BRD measured throughout the study period per 100 calf-days at risk was 0.54 (95% CI: 0.45–0.66), 0.60 (95%CI: 0.49–0.72), 1.33 (95% CI: 1.18–1.51), and 0.57 (95%CI: 0.47–0.69) for WRS, CRS, TUS2, and TUS3, respectively. The proportion of calves with a fecal score > 0 peaked between days 8–14, whereby 56.8% (n = 264), 19.0% (n = 88), 11.4% (n = 53), and 12.7% (n = 59) of calves had a score of 0, 1, 2, or 3, respectively. A similar pattern was evident for the hygiene score, with 37.6% (n = 170) of cases scoring >3 during days 8–14.

#### 3.2.2. New, Repeat, and Chronic Cases

**Figure 3:**
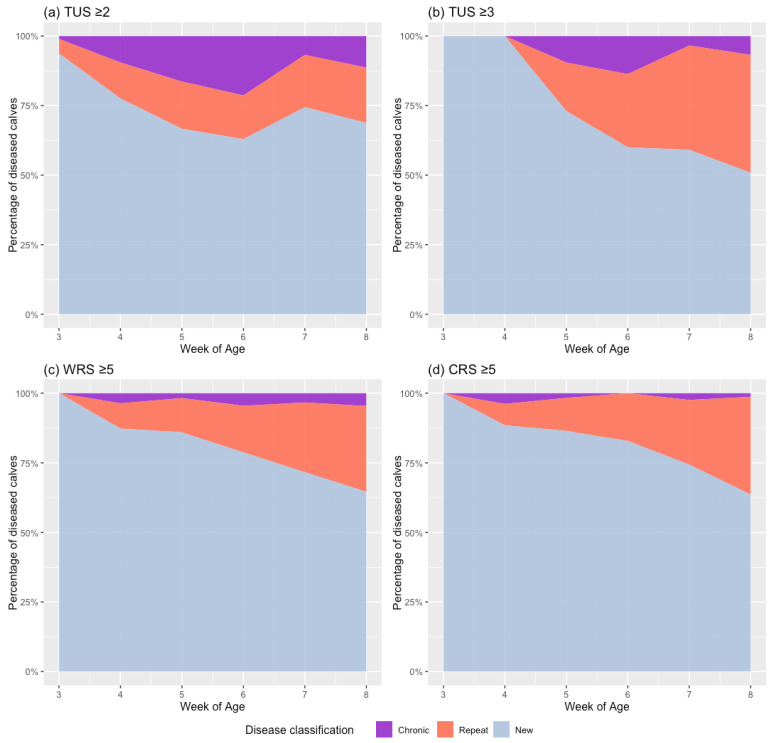
Percentage of calves with a case of bovine respiratory disease (BRD) defined as either new, repeat, or chronic, measured using thoracic ultrasonography (TUS) or Wisconsin (WRS) or California respiratory scoring (CRS) during the preceding 21-day period between 3 and 8 weeks of age. For TUS, the results were calculated using cut-offs of (**a**) ≥2 and (**b**) ≥3, whereas for (**c**) Wisconsin and (**d**) California respiratory scores, a cut-off of ≥5 was used.

The percentage of calves assigned to each case definition was analyzed overall and for each 21-day period over the duration of the study (Figure 3; Appendix A, Table A4 and Table A5). Regardless of the diagnostic method, a gradual reduction in the number of new cases and an increase in the number of repeat cases were observed with an increasing examination age. At the end of the study period, new cases accounted for 68.8% (n = 97), 50.8% (n = 60), 64.6% (n = 42), and 63.6% (n = 49) of all cases for TUS2, TUS3, WRS, and CRS, respectively. The proportions of calves assessed as having either repeated cases or chronic disease were affected by the assessment method used. Repeat cases represented 19.9% (n = 28) and 42.4% (n = 50) of all cases by days 35–56 measured using TUS2 and TUS3, respectively. The California and Wisconsin respiratory scoring system results were similar, with 30.8% (n = 20) and 35.1 (n = 27) of cases classified as repeats for WRS and CRS within the same timeframe, respectively. Chronic disease accounted for a greater percentage of cases for TUS measures, peaking at 21.3% (n = 38) and 13.7 (n = 13) for TUS2 and TUS3, compared to just 4.6% (n = 3) and 3.9% (n = 2) for WRS and CRS, respectively.

#### 3.2.3. BRD Subtype

**Figure 4:**
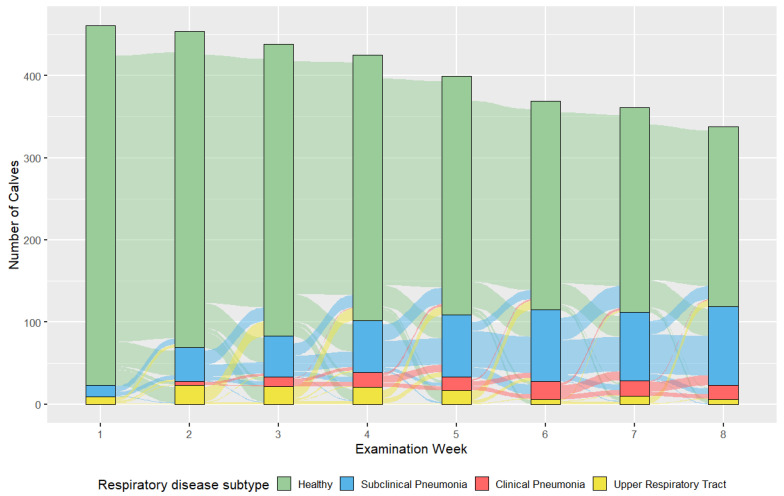
Alluvial plot of bovine respiratory disease (BRD) subtype defined using a Wisconsin respiratory score (WRS) threshold of ≥5 and a thoracic ultrasound score (TUS) of ≥2, performed during repeated examinations of 470–349 calves during an 8-week period from birth. BRD subtype was classified as either: (i) healthy (neither a BRD positive WRS (<5) or BRD positive lesions on TUS (<3 or <2)); (ii) localized to the upper respiratory tract (URT, a BRD positive WRS (≥5) in the absence of BRD positive lesions identified by TUS (<3 or <2)); (iii) subclinical pneumonia, present in the lower respiratory tract (LRT, a BRD negative WRS (<5) with BRD positive lesions identified on TUS (≥2 or ≥3)); or (iv) clinical pneumonia (both a BRD positive WRS (≥5) and BRD positive lesions identified on TUS (≥2 or ≥3)). The vertical columns represent each examination week. The colors within each column correlate to a TUS score depicted in the key at the bottom of the figure. The blocks of color passing between columns represent the proportion of calves moving between BRD subtypes throughout the study period.

**Figure A1:**
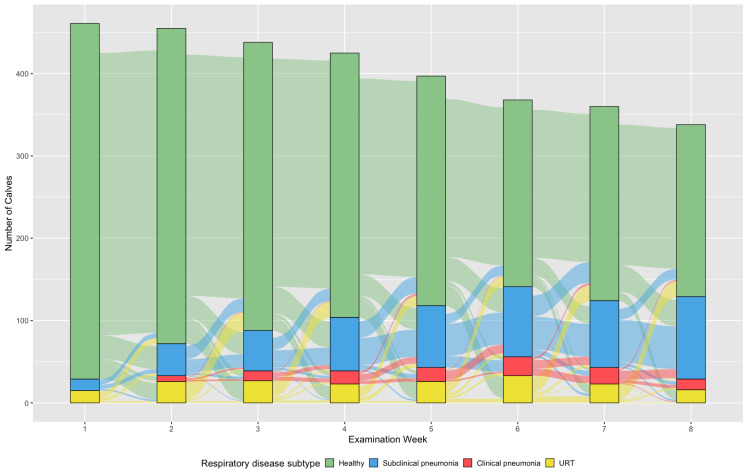
Alluvial plot of bovine respiratory disease (BRD) subtype^1^ defined using a California respiratory score (CRS) threshold of ≥5 and a thoracic ultrasound score (TUS) of ≥2, performed during repeated examinations of 470–349 calves during an 8-week period from birth. BRD subtype was classified as either (i) healthy (neither a significant CRS nor significant lesions on TUS; (ii) localized to the upper respiratory tract (a significant CRS in the absence of significant lesions identified by TUS; (iii) subclinical pneumonia (a non-significant CRS with significant lesions identified on TUS; or (iv) clinical pneumonia (both a significant CRS and significant lesions identified on TUS).

Healthy calves were classified as having a WRS < 5 and a TUS2 threshold of <2, implying a lobular consolidation < 1 cm^2^. There was a gradual reduction in the percentage of healthy calves during the study period, falling from 94.7% (445/470) in the first week of life to 64.8% (226/349) during 50–56 days of age (Figure 4 and Table 5). Similar associations were measured using CRS and TUS3 methods (Table 5, Appendix A, Figure A1 and Table A5).

The percentage of subclinical pneumonia cases was similar using CRS and WRS definitions. Both demonstrated a trend to increase with age, peaking during days 50–56 (CRS and TUS2; 29.5%; 103/349 and CRS and TUS3; 13.8%; 48/349). Calves were diagnosed with clinical pneumonia at between 0–6.3% and 0–4.8% of weekly examinations for lobular (TUS2) and lobar (TUS3) cut-offs, respectively. The percentage of upper respiratory tract localizations was greater for CRS than WRS, with a peak frequency during days 36–42, regardless of if a lobular (TUS2; 9.3%; 35/378) or lobar (TUS3, 13.5%; 51/279) cut-off was used to define URT cases.

### 3.3. Inferential Statistics

#### 3.3.1. Chronic and Repeat Cases

Modeling of chronic and repeat cases of disease was associated with a high degree of uncertainty, improved by the omission of growth and weight covariates from the analyses. Aside from farm, no covariates were significantly associated with chronic cases of disease when defined using either TUS2 or TUS3 thresholds (Supplement S1; Tables S1 and S2). However, when compared, calves experiencing lobar pneumonia (TUS ≥ 3) were significantly more likely to become chronic than calves with lobular pneumonia (TUS ≥ 2) (Pearson’s chi-squared test: χ^2^ = 136.37, df = 1, *p* < 0.001). After accounting for farm, an increase in the serum total protein concentration by 1 g/L was significantly associated (*p* = 0.04) with a 3% reduction in the odds of having a repeat case, defined using a TUS2 threshold. No similar association was retained using the TUS3 threshold. Calves destined for dairy production were significantly more likely to have a repeat case of consolidation measured using both TUS2 (OR 1.90, 95% CI 1.06–3.45) and TUS3 (OR 1.90, 95% CI 1.07–3.45) thresholds (Supplement S1; Tables S3 and S4). Similarly, a positive association was found for calves receiving treatment and the repeat classification (TUS2, OR 2.95, 95% CI 1.26–7.31; TUS3, OR 6.31, 95% CI 1.24–7.13).

#### 3.3.2. BRD Subtype

The final multivariate model for the outcome measure of BRD subtype included the significant predictors of farm, age, serum total protein, and fecal score (Table 6 and Table 7).

Nominal modeling demonstrated a significant between-farm variation. Each additional day of age was associated with a 5% increase in the likelihood of a (sub)clinical pneumonia diagnosis compared to healthy calves, and a 4% increase in the likelihood of a (sub)clinical pneumonia diagnosis compared to a URT localization. Increasing the serum total protein concentration was associated with a reduced odds of disease. The effect size was small but incremental, with each 1 g/L increase in titer, the likelihood of a healthy or URT diagnosis increased by 3% and 5% compared to (sub)clinical pneumonia, respectively. Elevated fecal score was associated with a greater likelihood of a (sub)clinical pneumonia phenotype, but no significant effect was identified when comparing pneumonic and URT localizations.

#### 3.3.3. TUS Score

The final ordinal GEE for the dependent variable of TUS score included the explanatory variables farm, age, weight, gender, Wisconsin respiratory score, and STP concentration.

Between-farm variation was marked. Each daily increase in age was associated with an 8% increase in the likelihood of classification into a higher TUS category. At any time, an increase in bodyweight of 1 kg was associated with a 4% reduced likelihood of scoring into a higher TUS category. Male calves were 69% more likely to score in higher TUS categories, whilst calves with an elevated WRS (≥5) were 5.6 times more likely to score within a higher TUS category. Increasing the STP concentration in week 1 was associated with a lower odds of classification into a higher TUS category. The effect size was small, but incremental, with each 1 g/L increase in titer associated with a 2% decrease (Table 8).

#### 3.3.4. Consolidation Depth

In the linear mixed-effects model for consolidation depth, gender, hygiene score, and purpose (dairy vs. beef) were removed from the model due to *p* > 0.20. The final multivariate model included the significant predictors of exam age (*p* = <0.001), STP (*p* = 0.04), WRS (≥5, *p* = <0.001), and fecal score (score 1).

Increasing age, fecal score, and a WRS ≥ 5 were all associated with an increased consolidation depth. A 1-day increase in exam age was, on average, associated with a 0.02 cm increase in consolidation depth, whereas an increase in the WRS score to ≥5 was associated with, on average, an increase in lung consolidation depth by 1.58 cm. Calves with a fecal score of 1 (*p* = 0.03) or 2 (*p* = 0.08) had an increased consolidation depth compared to calves scoring 0. Increasing STP in week 1 had a small but significant association with reduced consolidation depth, such that each 1 g/L increase was associated with an average reduction in consolidation depth of −0.01 cm (Table 9).

## 4. Discussion

This study comprehensively described and characterized the development and progression of BRD disease occurrence using repeated measurements of TUS and CRS within populations of preweaned calves born on UK dairy farms.

Longitudinal disease assessment allows the measurement of disease recurrence throughout the preweaning period, as well as the identification of temporal trends [50,51]. In this study, the highest disease frequencies were measured using TUS2, which identified 54% of the total calf population as cases in days 21–42 and, when measured throughout the study period, 1.6 disease occurrences per animal. Long-term reductions in average daily liveweight gain have been associated with TUS2 positivity, highlighting the importance of prompt recognition and effective treatment, even when not associated with concurrent clinical signs [25,30]. Interestingly, and similar to Urie et al. [52], the incidence rate of cases with a positive CRS during the study period remained relatively consistent. In comparison, using TUS, this study demonstrated a gradual increase in prevalence during the preweaning period (Table 4 and Figure 2). As the examination age increased, the percentage of cases classified as either repeat or chronic also increased, whereas new cases reduced (Figure 3).

The identification of chronic cases of respiratory disease is useful. Chronic lung consolidation has been associated with reduced prognoses of cure and survival, as well as reductions in growth rate and carcass weight [29,53]. At the herd level, the persistence of chronically infected individuals may also represent a source of infection to herd mates, especially in cases where *Mycoplasmopsis bovis* is implicated [54]. Repeat assessments can inform the management of these individuals and, since TUS identified a greater percentage of chronic cases than clinical scoring, it may be the superior diagnostic method for their detection. However, in this study, although examination data were provided to farmers and their veterinarians, the relative proportion of calves treated for respiratory disease during each week was low. This may be due to several reasons, including low confidence in treating animals without outward signs of disease and pressure to minimize antimicrobial therapy. It is possible that a low rate of antimicrobial therapy may have contributed to the high percentage of chronic cases identified on TUS in this study, emphasising the importance of identifying and managing TUS cases even in the absence of positive RS indicators.

Even when treated immediately, lung consolidation does not resolve in all cases [30]. Sequential TUS is therefore crucial to determine the progression of consolidation and effectiveness of therapy, especially since the disappearance of outward clinical signs of disease is not a good indicator of bacteriologic cure [55], whereas the correlation between TUS and pneumonia severity post-mortem is high [56]. Disease recurrence may be the result of impaired innate and adaptive immune responses following primary infection, or the failure of complete bacteriologic cure [30,57]. Our analyses found that increasing the STP concentration was protective against the occurrence of repeated lobular (TUS2) lung consolidation (OR 0.97, 95% CI 0.94–1.00). Any treatment (AB ± NSAID) was also significantly associated with repeat cases of disease (TUS2, OR 2.95, 95% CI 1.26–7.31), perhaps suggesting that a transient improvement occurred, followed by relapse, a pattern identified in similar studies [17,30] and more likely when the consolidation depth is greater [58]. It must also be acknowledged that repeat cases could represent a misclassification, associated with variations in symptoms during the disease process [33]. This is more likely for clinical scoring than TUS, due to its relative subjectivity. Nonetheless, operator error during TUS may also contribute to the false classification of repeat cases. Whilst no perfect diagnostic method for the antemortem diagnosis of BRD exists [10], the inter- and intra-operator repeatabilities of the diagnostic techniques used in this study were optimized through the use of a single, experienced, and specifically trained rater (GL) for all scoring throughout the study period.

This study also investigated factors associated with BRD subtype and lung consolidation. The findings reaffirm the role of passive immunity for the prevention of BRD [59,60]. An increasing STP concentration reduced the likelihood of progression from healthy or URT disease subtypes to (sub)clinical pneumonia, increased the likelihood of remaining in lower TUS categories, and, on average, reduced the depth of lung consolidation (Table 6, Table 7, Table 8 and Table 9). In contrast, an elevated WRS was associated with a greatly reduced likelihood of a low TUS score. This finding is consistent in suggesting that the ultrasonographic assessment of individuals with elevated RS values is necessary, although it lacks sensitivity [31,32], which may be further demonstrated by the visualization of the relatively low proportion of clinical cases versus subclinical cases of pneumonia found in this study (Figure 4 and Table 5). Modeling using GEE found that on average and compared to calves with a fecal score of 0, calves with fecal scores of 1 and 2 were 32% and 44% less likely to be healthy, respectively, compared with (sub)clinical phenotypes (Table 6). These scores were also associated with increases in consolidation depth (Table 9). Whilst a higher fecal score of 3 was not associated with other deleterious effects on the BRD subtype, this lack of association may be caused by the relatively small number of calves (12.7% (n = 59) at days 8–14) with high scores in this study.

BRD subtype classification may allow for precise treatment. Whereas the early detection and treatment of cases of subclinical pneumonia may restore growth potential [29], the identification of disease confined to the URT, including up to 14% of cases of in this study, is also useful. Disease associated with only rhinitis or pharyngitis may not necessarily require antimicrobial therapy, particularly if caused by a viral pathogen [20,42]. The results of this study suggest that URT disease does not consistently progress to clinical or subclinical pneumonia, and a proportion of cases are self-resolving (Figure 4 and Figure A1). Unfortunately, the relatively low proportion of URT cases in this study precluded the statistical testing of this hypothesis. Nonetheless, if true, this may present an opportunity to rationalize antimicrobial usage further. However, the extent of between-farm variation found by us and others suggests that the URT presentation may represent an oversimplification [30]. Other factors implicated in disease etiology, not limited to farm, age, and STP concentration, as found in this study, but also inclusive of other host, pathogen, and environmental aspects are implicated in disease progression (Table 7) [33,61,62]. Precise management of URT cases to ensure optimum welfare, health, and performance is crucial and requires more research [63].

Subclinical pneumonia prevalence is reported to be highly variable, and our findings are relatively lower than those published elsewhere. During a BRD outbreak in Belgian veal calves, 86.8% of cases were subclinical [29], whereas in commercial dairy herds in Ohio, 60.0% (139/233) and 70.9% (73/103) of cases were subclinical [25,64]. Nonetheless, the progression of lung consolidation we identified is not dissimilar to the findings elsewhere in the world. A study of 221 calves from a single dairy herd in Iran described an increase in lung consolidation throughout the preweaning period [65]. Likewise, a similar trend was observed in a study of two Canadian herds up to 13 weeks of age [66]. One difference between studies is that we selected a convenience sample of herds, rather than targeting herds with known BRD problems, potentially explaining why our measures of disease frequency were lower.

Limitations in the present study must be considered. Firstly, a number of calves were lost to follow up during the study period. This was expected, since our study design sought to enroll both replacement dairy heifers and stock-reared for beef production, to provide information regarding all calves originating from UK dairies. Although departure age was highly variable, calves destined for beef production frequently departed the farm of origin prior to the end of the study period. Secondly, the farms enrolled in this study represent a convenience sample selected based upon a willingness to participate, accessibility to the primary researcher, and calving pattern. On this basis, these may not be completely generalizable. However, participating farms were diverse in management practices, animal populations, and geographic location. Whilst they were not randomly selected, their characteristics remain broadly representative of the UK dairy herd population. Thirdly, this study performed both RS methods on a weekly basis. This may have contributed to the high proportion of cases of lung consolidation in the absence of a positive RS score, which occurred in up to 29.5% (103/349) of weekly examinations. In this study, these cases of subclinical pneumonia would have been undetected, representing an “unseen” loss for dairy producers associated with reduced productive and reproductive performances [22,67]. Nonetheless, both RS systems were designed to be performed more frequently than on a weekly basis, representing a limitation of this study since daily scoring was not possible.

## 5. Conclusions

This study describes the frequency of different BRD subtypes in calves born on UK dairy farms during the preweaning period. BRD varied by diagnostic technique and criteria, with a greater percentage of cases identified using TUS compared to respiratory scoring. Cases of subclinical pneumonia peaked during days 50–56, whereby 28.7% of calves were affected. Age, gender, STP concentration in week 1, weight, and fecal score were consistently associated with the repeated outcome measures of BRD subtype, TUS score, and consolidation depth. Stakeholders involved in preweaned calf management should consider the integrated use of respiratory scoring and TUS for the optimization of respiratory health.

## Figures and Tables

**Table 1:** Criteria and definitions of bovine respiratory disease subtype using combined clinical respiratory scoring and thoracic ultrasound.

Wisconsin or California Respiratory Score	Thoracic Ultrasound Score	Disease Subtype
<5	<2 (TUS2)	Healthy
	<3 (TUS3)	
≥5	<2 (TUS2)	Upper respiratory tract
	<3 (TUS3)	
<5	≥2 (TUS2)	Lower respiratory tract/subclinical pneumonia
	≥3 (TUS3)	
≥5	≥2 (TUS2)	Clinical pneumonia
	≥3 (TUS3)	

**Table 2:** Descriptive information of the 16 farms, including 476 calves enrolled in the study.

Farm	1	2	3	4	5	6	7	8	9	10	11	12	13	14	15	16
No. enrolled	29	23	30	30	31	33	25	37	31	30	41	27	23	30	37	19
Calving pattern ^1^	AYR	AYR	S & A	Spring	AYR	AYR	AYR	AYR	AYR	AYR	AYR	AYR	AYR	AYR	A	A
Predominant breed ^2^	H	H	HF X	HF X	H	HF	H	HF	H	H	HF	HF	H	HF	XB	H
Total no. milking cows	600	300	600	264	338	247	104	550	834	230	1350	200	255	189	210	200
Average herd 305 d yield	10,000	10,403	5900	5500	12,200	13,810	12,200	9000	11,331	11,079	11,888	9100	10,683	8500	7568	9800
Main colostrum feeding route	Suckle	Suckle	Suckle	Suckle	Bottle	Bottle	Bottle	Tube	Tube	Tube	Tube	Bottle	Suckle	Bottle	Suckle	Suckle
First colostrum feed volume	4				4	3	2.5–4	3	4.5	2.5	3	2.5		4		
Calf housing ^3^	GI	GI	GI	GI/GO	IO	IO	GI	GI/GO	GI	GI	II	GI	GI	GI/GO	GI	GI
Milk feeding method ^4^	Teat (A, B)	Teat (A, B)	Teat (G)	Teat (G)	Bottle/Teat (A)	Teat (B)	Teat (B)	Teat (B)	Teat (B)	Teat (B)	Teat (B)	Teat (G)	Teat (A, B)	Teat (B)	Teat (B)	Teat (G)
Milk source ^5^																
Replacements	MR	MR	MR	MR	MR	MR	WM	MR	MR	WM	MR	WM	MR	MR	MR	MR
Non-replacements	WM	WM	MR	MR	MR	WM	WM	MR	WM	WM	WM	WM	WM	MR	MR	WM
Peak daily milk DM (g) ^6^																
Replacements	750	1200	798	450	810	1080		900	1200		900		975	900	900	750
Non-replacements	750		399	450	1080			900						900	900	750

^1^ AYR = All-year round. Spring and Autumn calving herds calved during a discrete period within the described season. S = Spring; A = Autumn. ^2^ H = Holstein; HF = Holstein Friesian; XB H crossbreed cows, including British Friesian, Holstein Friesian, Brown Swiss, Swedish Red, and Norwegian Red. ^3^ GI = group inside, GO = group outside, IO = individual outside, and II = individual inside. ^4^ Teat feeders were either automated (A), attached to individual buckets (B), or attached to a group feeder (G). ^5^ Method of milk feeding for replacement and non-replacement stocks. WM = whole milk, MR = milk replacer. ^6^ Calculated for calves fed milk replacer only.

**Table 3:** Descriptive statistics of the study population, including the percentage [% (no.)] of calves by gender and breed, losses to follow up, and treatments recorded.

Exam No.	Age Range in Days	No. of Calves	Gender		Breed ^1^		Losses to Follow Up		Treatments ^3^	
			Male	Female	Dairy	Beef	Death	Total ^2^	NSAID	Antibiotic
1	1–7	476	23.3 (111)	76.7 (365)	56.9 (271)	43.1 (205)	0.4 (2)	2.3 (10)	1.5 (7)	2.7 (13)
2	8–14	464	23.1 (107)	76.9 (357)	57.5 (267)	42.5 (197)	0.9 (4)	2.6 (12)	0.6 (3)	1.7 (8)
3	15–21	448	97 (21.7)	78.3 (351)	59.4 (266)	40.6 (182)	0.7 (3)	2.9 (13)	0.7 (3)	0.7 (3)
4	22–28	441	20.4 (90)	79.6 (351)	60.3 (266)	39.7 (175)	0.2 (1)	1.4 (6)	1.3 (6)	0 (0)
5	29–35	411	19.7 (81)	80.3 (330)	64.5 (265)	35.5 (146)	0.7 (3)	6.6 (27)	1.4 (6)	1.6 (7)
6	36–42	378	18.8 (71)	81.2 (307)	66.4 (251)	33.6 (127)	0.5 (2)	8.2 (31)	3.5 (13)	1.3 (5)
7	43–49	377	19.4 (73)	80.6 (304)	68.4 (258)	31.6 (119)	0.3 (1)	0 (0)	1.3 (5)	0.2 (1)
8	50–56	349	19.5 (68)	80.5 (281)	67.9 (237)	32.1 (112)	0 0	8.0 (28)	1.7 (6)	1.1 (4)

^1^ Dairy calves included Holstein, Holstein Friesian, Brown Swiss, and Norwegian Red breeds. The sires of dairy-cross calves included Aberdeen Angus, Hereford, British Blue, and Limousin breeds. ^2^ Death, departure, weaning, or reasons unknown. ^3^ Described percentage represents the total number of doses during the week divided by the total number of calves enrolled during the week (total number of doses). Data were collected from treatment diaries at the end of the follow-up period.

**Table 4:** Prevalence and incidence of above-threshold measurements for thoracic ultrasonography scores collected during the weekly examination of calves born on dairy farms in South-west England from birth until weaning.

Days	No. of Calves	TUS2: TUS Score ≥ 2			TUS3: TUS Score ≥ 3		
		Prevalence (95% CI) ^3^	Incidence Rate ^1^ (95% CI) ^4^	% Incidence Risk ^2^ (95% CI) ^4^	Prevalence (95% CI) ^3^	Incidence Rate ^1^ (95% CI) ^4^	% Incidence Risk ^2^ (95% CI) ^4^
1–7	469	3.2 (1.6–4.8)	0.78 (0.44–1.30)	3.2 (1.8–5.3)	0.2 (0.0–0.6)	0.05 (0.00–0.29)	0.2 (0.0–1.2)
8–14	460	10.2 (7.5–13.0)	1.23 (0.87–1.7)	8.0 (5.7–8.1)	1.3 (0.3–2.3)	0.13 (0.04–0.34)	0.9 (0.2–2.2)
15–21	443	13.5 (10.4–16.7)	1.37 (0.96–1.86)	8.6 (6.1–11.8)	4.1 (2.2–5.9)	0.54 (0.30–0.88)	3.4 (1.9–5.6)
22–28	436	17.2 (13.7–20.7)	1.46 (1.00–2.00)	8.9 (6.4–12.2)	6.2 (3.9–8.5)	0.67 (0.40–1.05)	4.1 (2.4–6.5)
29–35	411	20.2 (16.3–24.1)	1.33 (0.91–1.90)	8.0 (5.5–11.3)	7.5 (5.0–10.1)	0.52 (0.28–0.89)	7.9 (0.02–0.06)
36–42	378	27.5 (23.0–32.0)	1.78 (1.30–2.48)	10.6 (7.6–14.4)	12.4 (9.1–15.8)	1.16 (0.77–1.73)	6.9 (4.5–10.1)
43–49	375	28.3 (23.7–32.8)	1.19 (0.77–1.73)	6.9 (4.5–10.2)	10.9 (7.7–14.1)	0.60 (0.31–1.01)	3.5 (1.8–5.9)
50–56	346	29.2 (24.4–34.0)	1.51 (1.00–2.10)	9.0 (6.1–12.7)	14.2 (10.5–17.8)	1.02 (0.62–1.53)	6.0 (3.8–9.3)

^1^ Calculated as cases per 100 calf-days at risk. ^2^ Calculated as: (number of new cases during each analysis period ÷ the number of calves at risk) × 100. ^3^ Calculated using Epitools [34]. ^4^ Calculated using MedCalc (MedCalc Software version 23.1.3, Ostend, Belgium).

**Table 5:** The number and percentage of calves identified with bovine respiratory disease during each week of age using a combined Wisconsin respiratory score (WRS) and thoracic ultrasound (TUS) scoring method ^1^.

Days	No. of Calves	TUS Score ≥ 2, Wisconsin Respiratory Score ≥ 5								TUS Score ≥ 3, Wisconsin Respiratory Score ≥ 5							
		Healthy		Upper Respiratory Tract		Subclinical Pneumonia		Clinical Pneumonia		Healthy		Upper Respiratory Tract		Subclinical Pneumonia		Clinical Pneumonia	
		No	%	No	%	No	%	No	%	No	%	No	%	No	%	No	%
1–7	470	445	94.7	10	2.1	15	3.2	0	0.0	459	97.7	10	2.1	1	0.2	0	0.0
8–14	461	391	84.8	23	5.0	41	8.9	6	1.3	423	91.8	32	6.9	5	1.1	1	0.2
15–21	443	356	80.4	22	5.0	54	12.2	11	2.5	390	88.0	35	7.9	12	2.7	6	1.4
22–28	436	333	76.4	21	4.8	63	14.4	19	4.4	372	85.3	34	7.8	15	3.4	15	3.4
29–35	409	297	72.6	17	4.2	78	19.1	17	4.2	341	83.4	31	7.6	31	7.6	6	1.5
36–42	378	259	68.5	6	1.6	90	23.8	23	6.1	311	82.3	19	5.0	31	8.2	17	4.5
43–49	375	260	69.3	10	2.7	86	22.9	19	5.1	306	81.6	23	6.1	32	8.5	14	3.7
50–56	349	226	64.8	6	1.7	100	28.7	17	4.9	279	79.9	17	4.9	42	12.0	11	3.2

^1^ Disease presentations were classified as either (i) healthy (neither a BRD positive WRS (<5) nor BRD positive lesions on TUS (<3 or <2)); (ii) localized to the upper respiratory tract (URT, a BRD positive WRS (≥5) in the absence of BRD positive lesions identified by TUS (<3 or <2)); (iii) subclinical pneumonia, present in the lower respiratory tract (LRT, a BRD negative WRS (<5) with BRD positive lesions identified on TUS (≥2 or ≥3)); or (iv) clinical pneumonia (both a BRD positive WRS (≥5) and BRD positive lesions identified on TUS (≥2 or ≥3)).

**Table 6:** Results of the nominal generalised estimating equation for bovine respiratory disease subtype ^1^ comparing healthy and (sub)clinical pneumonia localizations.

Variable		Estimate (SE)	95% CI	Odds Ratio ^2^	95% CI	*p*-Value ^3^
Intercept		−0.44 (0.64)	−1.69–0.82	1.55	0.44–5.44	0.49
Farm	1	Reference				
	2	1.61 (0.46)	0.7–2.52	0.20	0.08–0.50	<0.001 **
	3	3.54 (0.54)	2.48–4.6	0.03	0.01–0.08	<0.001 **
	4	2.99 (0.47)	2.06–3.91	0.05	0.02–0.13	<0.001 **
	5	3.02 (0.49)	2.06–3.98	0.05	0.02–0.13	<0.001 **
	6	3.55 (0.49)	2.59–4.51	0.03	0.01–0.08	<0.001 **
	7	4.22 (0.49)	3.27–5.18	0.01	0.01–0.04	<0.001 **
	8	0.95 (0.33)	0.3–1.61	0.38	0.2–0.74	<0.001 **
	9	−0.19 (0.29)	−0.76–0.39	1.21	0.68–2.14	0.52
	10	2.43 (0.37)	1.7–3.16	0.09	0.04–0.18	<0.001 **
	11	1.04 (0.3)	0.45–1.62	0.35	0.2–0.64	<0.001 **
	12	0.56 (0.37)	−0.18–1.29	0.57	0.28–1.20	0.14
	13	2.79 (0.53)	1.75–3.84	0.06	0.02–0.17	<0.001 **
	14	1.66 (0.38)	0.92–2.41	0.19	0.09–0.40	<0.001 **
	15	3.02 (0.5)	2.04–4.01	0.05	0.02–0.13	<0.001 **
	16	3.5 (0.53)	2.47–4.54	0.03	0.01–0.09	<0.001 **
Age		−0.05 (0)	−0.05–−0.04	1.05	1.04–1.06	<0.001 **
Serum total protein		0.03 (0.01)	0.01–0.04	0.97	0.96–0.99	0.01 *
Fecal score	0	Reference				
	1	−0.38 (0.14)	−0.66–−0.11	1.47	1.11–1.93	0.01 *
	2	−0.58 (0.23)	−1.02–−0.14	1.78	1.14–2.77	0.01 *
	3	−0.22 (0.23)	−0.68–0.24	1.24	0.79–1.97	0.35

^1^ BRD subtype is classified as either (i) healthy (neither a BRD positive WRS (<5) nor BRD positive lesions on TUS (<2)); (ii) localized to the upper respiratory tract (URT, a BRD positive WRS (≥5) in the absence of BRD positive lesions identified by TUS (<2)); (iii) subclinical pneumonia, present in the lower respiratory tract (LRT, a BRD negative WRS (<5) with BRD positive lesions identified on TUS (≥2)); or (iv) clinical pneumonia (both a BRD positive WRS (≥5) and BRD positive lesions identified on TUS (≥2)). For the nominal generalized estimating equation, subclinical pneumonia and clinical pneumonia were consolidated into a single category. ^2^ Odds ratio (OR) values > 1 are associated with a greater likelihood for (sub)clinical pneumonia, whereas OR values < 1 indicate a reduced likelihood of (sub)clinical pneumonia. ^3^ * = *p* < 0.05, and ** = *p* < 0.001.

**Table 7:** Results of the nominal generalised estimating equation for the bovine respiratory disease subtype ^1^ comparing upper respiratory tract (URT) to (sub)clinical pneumonia localizations.

Variable		Estimate (SE)	95% CI	Odds Ratio ^2^	95% CI	*p*-Value ^3^
Intercept		−3.94 (1.08)	−6.06–−1.82	51.26	6.15–427.63	<0.001 **
Farm	1	Reference	−1.74–1.15			
	2	−0.3 (0.74)	−0.09–3.1	1.35	0.32–5.71	0.69
	3	1.5 (0.81)	−1.74–1.84	0.22	0.05–1.09	0.06 ^#^
	4	0.05 (0.91)	−1.09–2.29	0.95	0.16–5.71	0.96
	5	0.6 (0.86)	−0.97–2.4	0.55	0.1–2.98	0.49
	6	0.72 (0.86)	0.17–3.34	0.49	0.09–2.64	0.40
	7	1.76 (0.81)	−0.46–1.73	0.17	0.04–0.84	0.03 *
	8	0.63 (0.56)	−1.58–0.56	0.53	0.18–1.58	0.26
	9	−0.51 (0.55)	−3.1–1.34	1.67	0.57–4.86	0.35
	10	−0.88 (1.13)	−2.13–0.53	2.41	0.26–22.26	0.44
	11	−0.8 (0.68)	−0.27–1.73	2.23	0.59–8.44	0.24
	12	0.73 (0.51)	0.96–3.67	0.48	0.18–1.30	0.15
	13	2.31 (0.69)	−1.09–1.38	0.10	0.03–0.38	<0.001 **
	14	0.14 (0.63)	−1.01–2.17	0.87	0.25–2.99	0.82
	15	0.58 (0.81)	1.66–4.1	0.56	0.11–2.76	0.48
	16	2.88 (0.62)	−0.05–−0.03	0.06	0.02–0.19	<0.001 **
Age		−0.04 (0.01)	0.02–0.08	1.04	1.03–1.05	<0.001 **
Serum total protein		0.05 (0.02)	−1.74–1.15	0.95	0.92–0.98	<0.001 **
Fecal score	0	Reference				
	1	0.13 (0.31)	−0.49–0.75	0.88	0.47–1.63	0.68
	2	−0.18 (0.42)	0.63–0.83	1.52	0.53–2.72	0.66
	3	0.55 (0.39)	1.32–1.74	6.40	0.27–1.24	0.16

^1^ BRD subtype is classified as either (i) healthy (neither a BRD positive WRS (<5) nor BRD positive lesions on TUS (<2)); (ii) localized to the upper respiratory tract (URT, a BRD positive WRS (≥5) in the absence of BRD positive lesions identified by TUS (<2)); (iii) subclinical pneumonia, present in the lower respiratory tract (LRT, a BRD negative WRS (<5) with BRD positive lesions identified on TUS (≥2)); or (iv) clinical pneumonia (both a BRD positive WRS (≥5) and BRD positive lesions identified on TUS (≥2)). For the nominal generalized estimating equation, subclinical pneumonia and clinical pneumonia were consolidated into a single category. ^2^ Odds ratio (OR) values > 1 are associated with a greater likelihood for (sub)clinical pneumonia, whereas OR values < 1 indicate a reduced likelihood of (sub)clinical pneumonia. ^3 #^ = *p* < 0.1, * = *p* < 0.05, and ** = *p* < 0.001.

**Table 8:** Results for the ordinal GEE model for the repeated measures of thoracic ultrasound score ^1^ of the study calves from birth until weaning.

Variable		Estimate (SE)	95% CI	Odds Ratio ^2^	95% CI	*p*-Value ^3^
Intercept	1	−0.61 (0.77)	−2.13–0.9	1.85	0.41–8.39	0.43
	2	1.02 (0.77)	−0.49–2.52	0.36	0.08–1.62	0.18
Farm	1	Reference				
	2	1.07 (0.52)	0.05–2.09	0.34	0.12–0.96	0.04 *
	3	3.69 (0.7)	2.32–5.06	0.03	0.01–0.10	<0.001 **
	4	2.76 (0.52)	1.74–3.77	0.06	0.02–0.17	<0.001 **
	5	2.63 (0.88)	0.91–4.34	0.07	0.01–0.4	<0.001 **
	6	2.23 (0.61)	1.03–3.43	0.11	0.03–0.36	<0.001 **
	7	3.55 (0.65)	2.28–4.82	0.03	0.01–0.10	<0.001 **
	8	0 (0.43)	−0.84–0.84	1.00	0.43–2.32	1.00
	9	−1.15 (0.41)	−1.94–−0.36	3.16	1.43–6.98	<0.001 **
	10	1.79 (0.51)	0.79–2.79	0.17	0.06–0.46	<0.001 **
	11	0.42 (0.35)	−0.27–1.11	0.66	0.33–1.31	0.23
	12	0.77 (0.45)	−0.11–1.65	0.46	0.19–1.12	0.09 ^#^
	13	2.94 (0.58)	1.81–4.06	0.05	0.02–0.16	<0.001 **
	14	1.3 (0.42)	0.48–2.12	0.27	0.12–0.62	<0.001 **
	15	2.44 (0.7)	1.07–3.81	0.09	0.02–0.34	<0.001 **
	16	0.3 (1.06)	−1.78–2.37	0.74	0.09–5.93	0.78
Age		−0.07 (0.01)	−0.09–−0.06	1.08	1.06–1.09	<0.001 **
Weight		0.04 (0.01)	0.01–0.06	0.96	0.94–0.99	<0.001 **
Gender	F	Reference				
	M	−0.53 (0.26)	−1.04–−0.01	1.69	1.01–2.84	0.05 *
Wisconsin respiratory score	<5	Reference				
	≥5	−1.72 (0.26)	−2.23–−1.22	5.61	3.38–9.30	<0.001 **
Serum total protein		0.02 (0.01)	0–0.05	0.98	0.95–1.00	0.03 *

^1^ A 6-point (0–5) score described by Ollivett et al. [38] was recategorized as: scores 0 and 1; 1, score 2; 2 and score ≥3; 3. ^2^ Odds ratio (OR) values >1 are associated with a greater likelihood of classification within a higher TUS category, whereas OR values < 1 indicate a reduced likelihood of classification within a higher TUS category. ^3 #^ = *p* < 0.1, * = *p* < 0.05, and ** = *p* < 0.001.

**Table 9:** Results for the linear mixed-effects model for the repeated measures consolidation depth ^1^ of the study calves from birth until weaning.

Variable		Estimate (SE)	95% CI	*p*-Value ^2^
Intercept		0.74 (0.32)	−0.02–1.07	0.06 *
Exam age		0.02 (0.00)	0.02–0.02	<0.001 **
Serum total protein (g/L)		−0.01 (0.00)	−0.01–0.00	0.04 *
Wisconsin respiratory score	<5	Baseline		
	≥5	1.58 (0.09)	1.40–1.77	<0.001 **
Fecal score	0	Baseline		
	1	0.14 (0.07)	0.01–0.28	0.03 *
	2	0.18 (0.10)	−0.02–0.38	0.08 ^#^
	3	−0.12 (0.12)	−0.35–0.11	0.31

^1^ Maximum depth of consolidation was measured in centimeters as a straight line perpendicular to the pleural line. ^2 #^ = *p* < 0.1, * = *p* < 0.05, and ** = *p* < 0.001.

## Data Availability

Data are available on request due to privacy restrictions.

## Supplementary Materials

The following supporting information can be downloaded at: https://www.mdpi.com/article/10.3390/ani15030360/s1. Table S1. Results of binary logistic regression analysis for chronic disease defined using a TUS2 threshold; Table S2. Results of binary logistic regression analysis for chronic disease defined using a TUS3 threshold; Table S3. Results of binary logistic regression analysis for repeat disease defined using a TUS2 threshold; Table S4. Results of binary logistic regression analysis for repeat disease defined using a TUS3 threshold.

## Appendix A

**Table A1:** Components and weighting of the Wisconsin and California clinical respiratory scoring methods. For both methods, a significant score was defined at a total score of ≥5.

Clinical Sign	California Respiratory Score ^1^			Wisconsin Respiratory Score ^2^				
	Weighting		Criteria ^3^	Weighting				Criteria ^3^
	Normal	Abnormal		Normal	Abnormal			
					Mi ^3^	Mo ^3^	Sev ^3^	
Ocular discharge	0	2	N = Normal Ab = Spontaneous cough	0	1	2	3	N = Normal Mi = Small amount of ocular discharge Mo = Moderate amount of bilateral discharge S = Heavy ocular discharge
Nasal discharge	0	4	N = Normal Ab = Any discharge	0	1	2	3	N = Serous discharge Mi = Unilateral cloudy discharge Mo = Bilateral, cloudy, or excessive discharge S = Copious mucopurulent discharge
Ear droop or head tilt	0	5	N = Normal, an ear flick, or head shake Ab = Ear droop or head tilt	0	1	2	3	N = Normal Mi = Ear flick or head shake Mo = Slight unilateral droop S = Head tilt or bilateral droop
Cough	0	2	N = None Ab = Any	0	1	2	3	N = Absent Mi = Inducible single cough Mo = Inducible repeated or occasional spontaneous cough S = Repeated spontaneous cough
Breathing	0	2	N = Normal respiration Ab = Abnormal respiration	NA	NA	NA	NA	
Temperature	0	2	N = <39.2 °C Ab = ≥39.2 °C	0	1	2	3	N = 37.8–38.2 °C Mi = 38.3–38.8 °C Mo = 38.9−39.3 °C S = ≥39.4 °C

^1^ As described by McGuirk and Peek [14]. ^2^ As described by Love et al. [13]. ^3^ For the California respiratory score symptoms were defined as normal (N) or abnormal (Ab), whereas for the Wisconsin respiratory score symptoms were classified as either normal (N), mild (Mi), moderate (Mo) or severe (S).

**Table A2:** The ordinal 6-point thoracic ultrasound (TUS) scoring method and the TUS2 and TUS3 criteria, and their definitions.

Thoracic Ultrasound Score ^1^	TUS2	TUS3	Defininition
0	−	−	Normal aerated lung
1	−	−	Diffuse comet tails
2	+	−	Lobular pneumonia: discrete areas of consolidation ≥ 1 cm^2^ within an otherwise aerated lung
3	+	+	Lobar pneumonia: consolidation of 1 entire lung lobe
4	+	+	Lobar pneumonia: consolidation of 2 entire lung lobes
5	+	+	Lobar pneumonia: consolidation of ≥3 entire lung lobes

^1^ As described by Ollivett et al. [41].

**Table A3:** Comparison of the frequency of new, chronic, and repeat cases of respiratory disease as a percentage of the total (%) and diseased (%t) calf population during each time period measured using thoracic ultrasonography (TUS) during a 21-day period between birth and 8 weeks of age.

Days	No. of Calves			TUS: Score ≥ 2									TUS: Score ≥ 3						
		New ^1^			Chronic ^2^			Repeat ^3^			New ^1^			Chronic ^2^			Repeat ^3^		
		No.	%	%t	No.	%	%t	No.	%	%t	No.	%	%t	No.	%	%t	No.	%	%t
1–21	398	90	22.6	93.8	1	0.3	1.0	5	1.3	5.2	20	5.0	100	0	0.0	0.0	0	0.0	0.0
7–28	383	114	29.8	77.6	14	3.7	9.5	19	5.0	12.9	37	9.7	100	0	0.0	0.0	0	0.0	0.0
14–36	355	110	31.0	66.7	27	7.6	16.4	28	7.9	17.0	46	13.0	73	6	1.7	9.5	11	3.1	17.5
21–42	331	112	33.8	62.9	38	11.5	21.3	28	8.5	15.7	57	17.2	60	13	3.9	13.7	25	7.6	26.3
28–49	312	99	31.7	74.4	9	2.9	6.8	25	8.0	18.8	52	16.7	59.1	3	1.0	3.4	33	10.6	37.5
35–56	298	97	32.6	68.8	16	5.4	11.3	28	9.4	19.9	60	20.1	50.8	8	2.7	6.8	50	16.8	42.4

^1^ New cases of disease were defined as cases where an above-threshold cut-off score was measured in an animal without any previous history of disease. ^2^ Chronic cases of disease were defined as cases where an above-threshold cut-off score was found in an animal for ≥21 days. ^3^ Repeat cases were defined as cases where an above-threshold cut-off score was found in an animal with a prior above cut-off score but a below-threshold cut-off score during the preceding examination.

**Table A4:** Comparison of the frequency of new, chronic, and repeat cases of respiratory disease as a percentage of the total (%) and diseased (%t) calf population during each time period measured using Wisconsin and California respiratory scoring during a 21-day period between birth and 8 weeks of age.

Days	No. of Calves			Wisconsin Respiratory Score: ≥5									California Respiratory Score: ≥5						
		New ^1^			Chronic ^2^			Repeat ^3^			New ^1^			Chronic ^2^			Repeat ^3^		
		No.	%	%t	No.	%	%t	No.	%	%t	No.	%	%t	No.	%	%t	No.	%	%t
1–21	398	30	7.5	100.0	0	0.0	0.0	0	0.0	30	33	8.3	100.0	0	0.0	0.0	0	0.0	0.0
7–28	383	48	12.5	87.3	2	0.5	3.6	5	1.3	48	46	12.0	88.5	2	0.5	3.9	4	1.0	7.7
14–36	355	49	13.8	86.0	1	0.3	1.8	7	2.0	49	51	14.4	86.4	1	0.3	1.7	7	2.0	11.9
21–42	331	52	15.7	78.8	3	0.9	4.6	11	3.3	52	63	19.0	82.9	0	0.0	0.0	13	3.9	17.1
28–49	312	43	13.8	71.7	2	0.6	3.3	15	4.8	43	61	19.6	74.4	2	0.6	2.4	19	6.1	23.2
35–56	298	42	14.1	64.6	3	1.0	4.6	20	6.7	42	49	16.4	63.6	1	0.3	1.3	27	9.1	35.1

^1^ New cases of disease were defined as cases where an above-threshold cut-off score was measured in an animal without any previous history of disease. ^2^ Chronic cases of disease were defined as cases where an above-threshold cut-off score was found in an animal for ≥21 days. ^3^ Repeat cases were defined as cases where an above-threshold cut-off score was found in an animal with a prior above-threshold cut-off score but a below-threshold cut-off score during the preceding examination.

**Table A5:** The number and percentage of calves identified with bovine respiratory disease, refined by subtype, during each week of age ^1^.

Days	No. of Calves	TUS: Score ≥ 2, California Respiratory Score ≥ 5								TUS: Score ≥ 3, California Respiratory Score ≥ 5							
		Healthy		Upper Respiratory Tract		Subclinical Pneumonia		Clinical Pneumonia		Healthy		Upper Respiratory Tract		Subclinical Pneumonia		Clinical Pneumonia	
		No	%	No	%	No	%	No	%	No	%	No	%	No	%	No	%
1–7	470	439	93.4	16	3.4	15	3.2	0	0.0	453	96.4	16	3.4	1	0.2	0	0.0
8–14	461	388	84.2	26	5.6	40	8.7	7	1.5	417	90.5	38	8.2	6	1.3	0	0.0
15–21	443	351	79.2	27	6.1	53	12.0	12	2.7	385	86.9	40	9.0	13	2.9	5	1.1
22–28	436	331	75.9	23	5.3	65	14.9	17	3.9	367	84.2	39	8.9	19	4.4	11	2.5
29–35	409	287	70.2	27	6.6	77	18.8	18	4.4	326	79.7	46	11.2	29	7.1	8	2.0
36–42	378	230	60.8	35	9.3	89	23.5	24	6.3	279	73.8	51	13.5	30	7.9	18	4.8
43–49	375	246	65.6	24	6.4	84	22.4	21	5.6	290	77.3	39	10.4	30	8.0	16	4.3
50–56	349	216	61.9	16	4.6	103	29.5	14	4.0	265	75.9	31	8.9	48	13.8	5	1.4

^1^ Disease presentations were classified as either (i) healthy (neither a significant California respiratory score (<5) nor significant lesions on TUS (<3 or <2)); (ii) localized to the upper respiratory tract (a significant California respiratory score (≥5) in the absence of significant lesions identified by TUS (<3 or <2)); (iii) subclinical pneumonia (a non-significant California respiratory score (<5) with significant lesions identified on TUS (≥2 or ≥3)); or (iv) clinical pneumonia (both a significant California respiratory score (≥5) and significant lesions identified on TUS (≥2 or ≥3)).
