# Supplementary material for: Development and Progression of Bovine Respiratory Disease Measured Using Clinical Respiratory Scoring and Thoracic Ultrasonography in Preweaned Calves on Dairy Farms in the United Kingdom: A Prospective Cohort Study
Source: Animals (Basel). 2025 Jan 27;15(3):360. doi: 10.3390/ani15030360 (PMC11816342; doi:10.3390/ani15030360)
Supplement: Supplementary file 1 [file animals-15-00360-s001.zip › animals-3356859-supplementary.pdf]

Table S1. Results of binary logistic regression analysis for chronic disease defined using a TUS2 threshold. A chronic case was defined as a thoracic ultrasound score ≥2 measured in an individual for a duration of ≥21 days (equivalent to a minimum of three consecutive examinations).

Variable		Estimate (SE)	95% CI	Odds ratio	95% CI	*p*-value[2]
Intercept		-0.28 (0.53)	-1.33 - 0.76	0.75	0.27 - 2.15	0.59
Farm	1	Reference				
	2	-0.97 (0.64)	-2.27 - 0.26	0.38	0.1 - 1.3	0.13
	3	-3.14 (1.14)	-6.14 - -1.25	0.04	0 - 0.29	0.01**
	4	-3.53 (1.09)	-6.49 - -1.76	0.03	0 - 0.17	< 0.001**
	5	-18.76 (1203.64)	-480.69 - 20.75	0.00	0 – 1.03 x10^{9}	0.99
	6	-3.43 (0.97)	-5.7 - -1.76	0.03	0 - 0.17	< 0.001**
	7	-18.35 (1356.5)	-528.15 - 28.15	0.00	0 - 1.68 x10^{12}	0.99
	8	-1.86 (0.66)	-3.21 - -0.62	0.16	0.04 - 0.54	< 0.001**
	9	0.9 (0.65)	-0.35 - 2.21	2.46	0.7 - 9.14	0.17
	10	-2.69 (0.84)	-4.66 - -1.21	0.07	0.01 - 0.3	< 0.001**
	11	-1.09 (0.54)	-2.18 - -0.05	0.34	0.11 - 0.95	0.04*
	12	-0.89 (0.6)	-2.1 - 0.28	0.41	0.12 - 1.32	0.14
	13	-18.67 (1449.25)	-570.7 - 29.66	0.00	0 – 7.61 x10^{12}	0.99
	14	-1.86 (0.69)	-3.33 - -0.57	0.16	0.04 - 0.57	0.01*
	15	-3.66 (1.1)	-6.62 - -1.89	0.03	0 - 0.15	< 0.001**
	16	-3.2 (1.1)	-6.17 - -1.41	0.04	0 - 0.24	< 0.001**
Purpose	Beef	Reference				
	Dairy	0.59 (0.35)	-0.08 - 1.3	1.81	0.92 - 3.69	0.09[#]
Treatment		1.00 (0.49)	0.05 - 2.03	2.71	1.06 - 7.6	0.04*

[2] # = p <0.1, * = p <0.05, ** = p <0.001

Table S2. Results of binary logistic regression analysis for chronic disease defined using a TUS3 threshold. A chronic case was defined as a thoracic ultrasound score ≥3 measured in an individual for a duration of ≥21 days (equivalent to a minimum of three consecutive examinations).

Variable		Estimate (SE)	95% CI	Odds ratio	95% CI	*p*-value[2]
Intercept		-1.2 (0.47)	-2.21 - -0.35	0.30	0.11 - 0.7	0.01*
Farm	1	Reference				
	2	-0.53 (0.78)	-2.2 - 0.95	0.59	0.11 - 2.59	0.50
	3	-19.36 (3237.11)	-1070.41 - 121.79	0.00	0 - 7.78 $x10^{52}$	1.00
	4	-19.36 (3237.11)	-938.82 - 145.79	0.00	0 – 2.07 $x10^{63}$	1.00
	5	-19.58 (3289.81)	-988.35 - 142	0.00	0 – 4.66 $x10^{61}$	1.00
	6	-19.9 (3008.27)	-905.76 - 127.85	0.00	0 – 3.35 $x10^{55}$	0.99
	7	-19.36 (3697.04)	-1082.98 - 166.79	0.00	0 – 2.73 $x10^{72}$	1.00
	8	-1.54 (0.84)	-3.31 - 0.04	0.21	0.04 - 1.04	0.07[#]
	9	0.61 (0.65)	-0.65 - 1.92	1.85	0.52 - 6.84	0.34
	10	-19.36 (3237.11)	-1142.84 - 108.57	0.00	0 – 1.42 $x10^{47}$	1.00
	11	-19.36 (2839.13)	-1078.77 - 79.33	0.00	0 – 2.85 $x10^{34}$	0.99
	12	-0.88 (0.77)	-2.53 - 0.58	0.42	0.08 - 1.79	0.26
	13	-19.36 (3964.63)	-1186.85 - 175.36	0.00	0 – 1.44 $x10^{76}$	1.00
	14	-2.16 (1.12)	-5.14 - -0.3	0.11	0.01 - 0.74	0.05[#]
	15	-19.36 (2914.85)	-1095.95 - 83.99	0.00	0 – 2.99 $x10^{36}$	0.99
	16	-19.36 (4067.63)	-1314.6 - 162.65	0.00	0 – 4.33 $x10^{70}$	1.00
Treatment		1 (0.62)	-0.16 - 2.33	2.72	0.85 - 10.28	0.11

[2] # = p <0.1, * = p <0.05, ** = p <0.001

Table S3. Results of binary logistic regression analysis for repeat disease defined using a TUS2 threshold. A repeat case was defined as a thoracic ultrasound score ≥2 followed by at least one clinically normal score (i.e. <2) and then a subsequent score ≥2.

Variable		Estimate (SE)	95% CI	Odds ratio	95% CI	*p*-value[2]
Intercept		3.79 (1.23)	1.47 - 6.38	44.29	4.33 - 589.92	< 0.001**
Farm	1	Reference				
	2	-3.19 (0.89)	-5.24 - -1.61	0.04	0.01 - 0.2	< 0.001**
	3	-5.12 (1.29)	-8.33 - -2.96	0.01	0 - 0.05	< 0.001**
	4	-3.93 (0.92)	-6.04 - -2.3	0.02	0 - 0.1	< 0.001**
	5	-20.07 (717.48)	-308 - -20.69	0.00	0 - 0	0.98
	6	-4.96 (1)	-7.22 - -3.21	0.01	0 - 0.04	< 0.001**
	7	-5 (1.29)	-8.22 - -2.84	0.01	0 - 0.06	< 0.001**
	8	-3.57 (0.87)	-5.58 - -2.03	0.03	0 - 0.13	< 0.001**
	9	-0.82 (0.92)	-2.87 - 0.89	0.44	0.06 - 2.45	0.37
	10	-4.6 (0.97)	-6.81 - -2.9	0.01	0 - 0.06	< 0.001**
	11	-2.23 (0.81)	-4.15 - -0.82	0.11	0.02 - 0.44	0.01*
	12	-2.55 (0.85)	-4.53 - -1.04	0.08	0.01 - 0.35	< 0.001**
	13	-5.41 (1.27)	-8.61 - -3.29	0.00	0 - 0.04	< 0.001**
	14	-2.58 (0.86)	-4.57 - -1.07	0.08	0.01 - 0.34	< 0.001**
	15	-4.36 (0.92)	-6.46 - -2.75	0.01	0 - 0.06	< 0.001**
	16	-5.48 (1.27)	-8.68 - -3.38	0.00	0 - 0.03	< 0.001**
Serum total protein (g/L)		-0.03 (0.02)	-0.06 - 0	0.97	0.94 - 1	0.04*
Purpose	Beef	Reference				
	Dairy	0.64 (0.3)	0.06 - 1.24	1.90	1.06 - 3.45	0.03*
Treatment		3.79 (1.23)	1.47 - 6.38	2.95	1.26 - 7.31	0.01*

[2] # = p <0.1, * = p <0.05, ** = p <0.001

Table S4. Results of binary logistic regression analysis for repeat disease defined using a TUS3 threshold. A repeat case was defined as a thoracic ultrasound score ≥3 followed by at least one clinically normal score (i.e. <3) and then a subsequent score ≥3.

Variable		Estimate (SE)	95% CI	Odds ratio	95% CI	*p*-value[2]
Intercept		1.84 (0.79)	0.48 - 3.74	6.31	1.61 - 42.21	0.02*
Farm	1	Reference				
	2	-3.13 (0.89)	-5.17 - -1.55	0.04	0.01 - 0.21	< 0.001**
	3	-5.27 (1.28)	-8.48 - -3.12	0.01	0 - 0.04	< 0.001**
	4	-4.19 (0.91)	-6.29 - -2.6	0.02	0 - 0.07	< 0.001**
	5	-19.92 (724.92)	-305.76 - -22.01	0.00	0 - 0	0.98
	6	-4.79 (0.99)	-7.04 - -3.05	0.01	0 - 0.05	< 0.001**
	7	-5.01 (1.29)	-8.22 - -2.86	0.01	0 - 0.06	< 0.001**
	8	-3.5 (0.87)	-5.51 - -1.97	0.03	0 - 0.14	< 0.001**
	9	-0.82 (0.92)	-2.87 - 0.89	0.44	0.06 - 2.45	0.37
	10	-4.4 (0.96)	-6.6 - -2.72	0.01	0 - 0.07	< 0.001**
	11	-2.36 (0.81)	-4.28 - -0.96	0.09	0.01 - 0.38	< 0.001**
	12	-2.54 (0.85)	-4.51 - -1.04	0.08	0.01 - 0.35	< 0.001**
	13	-5.22 (1.27)	-8.41 - -3.12	0.01	0 - 0.04	< 0.001**
	14	-2.81 (0.85)	-4.79 - -1.31	0.06	0.01 - 0.27	< 0.001**
	15	-4.34 (0.91)	-6.44 - -2.74	0.01	0 - 0.06	< 0.001**
	16	-5.38 (1.26)	-8.56 - -3.28	0.00	0 - 0.04	< 0.001**
Purpose	Beef	Reference				
	Dairy	0.64 (0.3)	0.07 - 1.24	1.90	1.07 - 3.45	0.03*
Treatment		1.06 (0.44)	0.22 - 1.96	6.31	1.24 - 7.13	0.02*

[2] # = p <0.1, * = p <0.05, ** = p <0.001
